# Mutations leading to ceftolozane/tazobactam and imipenem/cilastatin/relebactam resistance during *in vivo* exposure to ceftazidime/avibactam in *Pseudomonas aeruginosa*

**DOI:** 10.1128/spectrum.02312-24

**Published:** 2025-02-11

**Authors:** Glenn J. Rapsinski, Alecia B. Rokes, Daria Van Tyne, Vaughn S. Cooper

**Affiliations:** 1 Department of Microbiology and Molecular Genetics, University of Pittsburgh School of Medicine, Pittsburgh, Pennsylvania, USA; 2 Division of Infectious Disease, Department of Pediatrics, University of Pittsburgh School of Medicine, Pittsburgh, Pennsylvania, USA; 3 Division of Infectious Diseases, Department of Medicine, University of Pittsburgh School of Medicine, Pittsburgh, Pennsylvania, USA; 4 Center for Evolutionary Biology and Medicine, University of Pittsburgh School of Medicine, Pittsburgh, Pennsylvania, USA; Universidad de Buenos Aires, Buenos Aires, Argentina

**Keywords:** antibiotic resistance, *Pseudomonas aeruginosa*, drug resistance evolution

## Abstract

Identifying resistance mechanisms to novel antimicrobials informs treatment strategies during infection and antimicrobial development. Studying resistance that develops during the treatment of an infection can provide the most clinically relevant mutations conferring resistance, but cross-sectional studies frequently identify multiple candidate resistance mutations without resolving the driver mutation. We performed whole-genome sequencing of longitudinal *Pseudomonas aeruginosa* from a patient whose *P. aeruginosa* developed imipenem/cilastatin/relebactam and ceftolozane/tazobactam resistance during ceftazidime/avibactam treatment. This analysis determined new mutations that arose in isolates resistant to both imipenem/cilastatin/relebactam and ceftolozane/tazobactam. Mutations in penicillin-binding protein 3 *ftsI,* the MexAB-OprM repressor *nalD*, and a virulence regulator *pvdS* were found in resistant isolates. Importantly, drug efflux was not increased in the resistant isolate compared to the most closely related susceptible isolates. We conclude that mutations in peptidoglycan synthesis genes can alter the efficacy of multiple antimicrobials.

**IMPORTANCE:**

Antibiotic resistance is a significant challenge for physicians trying to treat infections. The development of novel antibiotics to treat resistant infections has not been prioritized for decades, limiting treatment options for infections caused by many high-priority pathogens. Cross-resistance, when one mutation provides resistance to multiple antibiotics, is most problematic. Mutations that cause cross-resistance need to be considered when developing new antibiotics to guide developers toward drugs with different targets, and thus a better likelihood of efficacy. This work was undertaken to determine the mutation that caused resistance to three antibiotics for highly resistant *Pseudomonas aeruginosa* infection treatment while the bacteria were exposed to only one of these agents. The findings provide evidence that drug developers should endeavor to find effective antibiotics with new targets and that medical providers should utilize medications with different mechanisms of action in bacteria that have become resistant to even one of these three agents.

## OBSERVATION

Antimicrobial resistance (AMR) is a public health crisis costing over a million lives and billions of dollars in healthcare costs each year (1). Cross-resistance, resistance to multiple antimicrobials from a single bacterial adaptation, further complicates AMR (2). Cross-resistance can occur through non-specific mechanisms, such as multi-drug efflux (3), and class-specific mechanisms (4). Nosocomial pathogens, like *Pseudomonas aeruginosa,* are primarily responsible for the AMR burden (1). *P. aeruginosa*, a gram-negative opportunistic pathogen, can cause ventilator-associated pneumonia, burn infections, sepsis, and chronic infection in people with lung diseases like cystic fibrosis (5, 6). Persistent infections lead to frequent antibiotic exposures and AMR emergence.

Ceftazidime/avibactam (CZA), ceftolozane/tazobactam (C/T), and imipenem/cilastatin/relebactam (IMI/REL) are antimicrobials developed for treating multidrug-resistant (MDR) infections. These beta-lactam/beta-lactamase inhibitor combinations (BL/BLI) inhibit the transpeptidase activity of penicillin-binding proteins (PBPs) in the cell wall and block beta-lactam-degrading enzymes (7, 8). While studies have determined many possible mechanisms of resistance to these agents (9–11), multiple coexisting mutations in isolates muddle conclusions about which mutations are necessary for resistance. We present the evolution of coincident CZA, C/T, and IMI/REL resistance during CZA treatment of one infection and determine the resistance-driving bacterial mutations to inform treatment and drug development for resistant organisms.

Serial *P. aeruginosa* isolates cultured from a patient with a tracheostomy tube and a peritoneal infection were collected from the clinical microbiology lab of UPMC Children’s Hospital of Pittsburgh (IRB: STUDY21080186). Early isolates were from tracheal aspirate specimens, and CZA treatment isolates were from a peritoneal infection. Ten isolates from eight sample dates were tested. The minimum inhibitory concentration (MIC) for CZA, C/T, and IMI/REL was determined using Liofilchem MIC Test Strips. DNA was extracted using DNeasy Blood and Tissue Kit (Qiagen). Short read whole-genome sequencing was performed to a minimum depth of coverage of 1.3 million reads by SeqCoast Genomics (Portsmouth, NH, USA), reads were trimmed with Trimmomatic, and genomes were assembled using SPAdes (12, 13). Genome annotation was performed using Prokka, and variant calling was completed using breseq version 0.38 (14, 15). A phylogenetic tree was created using RAxML version 8.2.11 with the rapid bootstrap algorithm, 2,421 random seed, and 1,000 runs using core genome assemblies created with Panaroo (16, 17) and visualized using iTOL (18).

**Fig 1:**
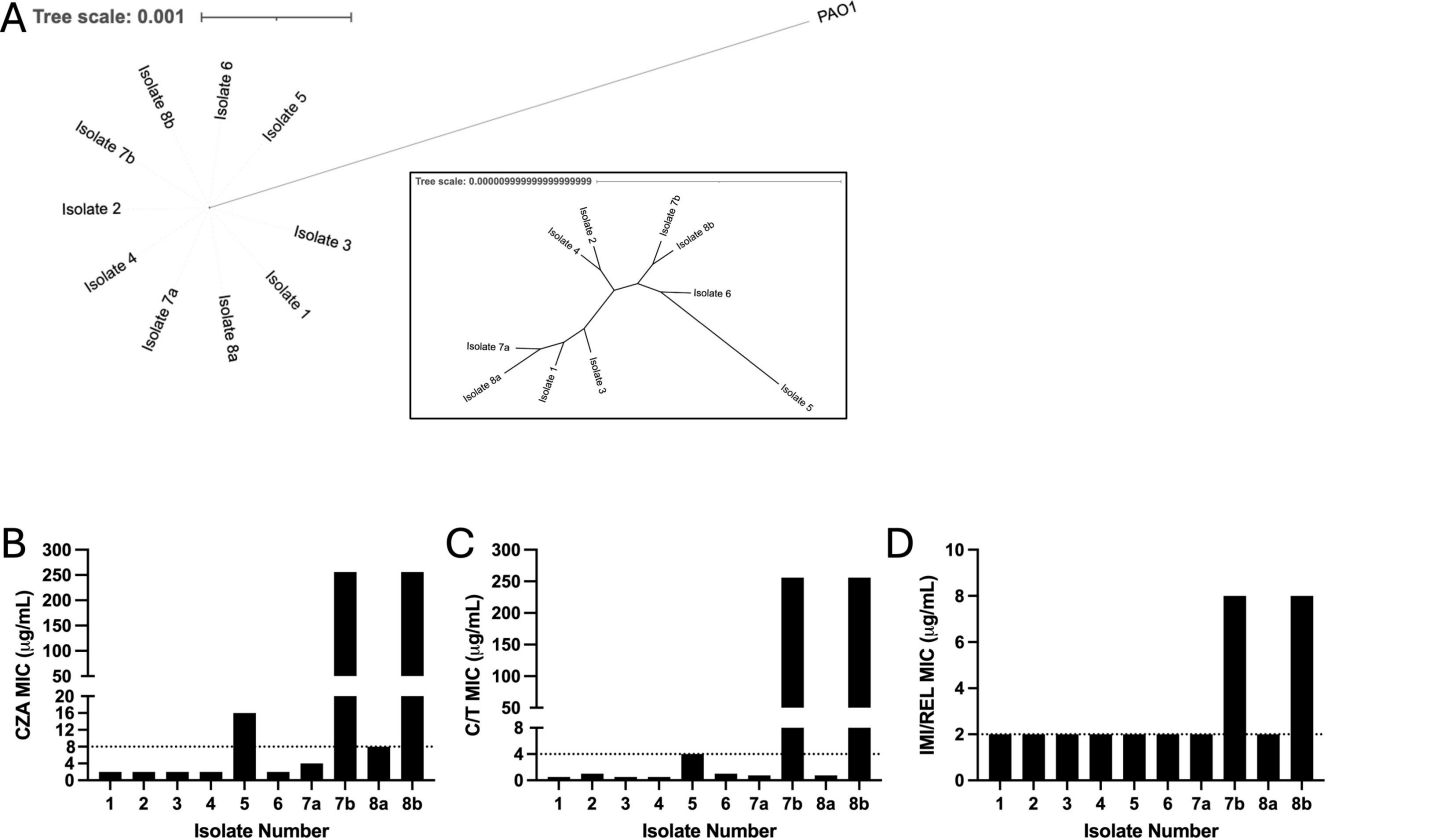
Isolates were all genetically linked and developed ceftazidime/avibactam (CZA), ceftolozane/tazobactam (C/T), imipenem/relebactam (IMI/REL) resistance over time. (**A**) Phylogenetic tree demonstrating genetic relatedness of longitudinal isolates collected over time. Number indicates sample date, and letter differentiates isolates from the same date. Inset shows zoom in of the isolates without PAO1 outgroup. (**B–D**) MIC for isolates to (**B**) CZA, (**C**) C/T, and (**D**) IMI/REL. Dashed lines indicate susceptibility breakpoints according to CLSI criteria.

The draft genome assembly of the ancestor (Isolate 1) was of good quality, consisting of 109 contigs with 6.867 Mbp found in contigs ≥ 10,000 bp and an *N*_50_ of 172,009. All isolates were monophyletic with a maximum of 10 single nucleotide polymorphisms (SNPs) between them (Fig. 1A). The only strains considered resistant by Clinical Laboratory Standards Institute criteria were strains 7b and 8b with CZA and C/T MIC of >256 and 8 µg/mL for IMI/REL (Fig. 1B, C, and D). While “b” isolates differed from their date-paired “a” isolate by colony morphology and resistance pattern, phylogenetic analysis confirmed that they were descendants of the initial strain (Fig. 1A).

**Fig 2:**
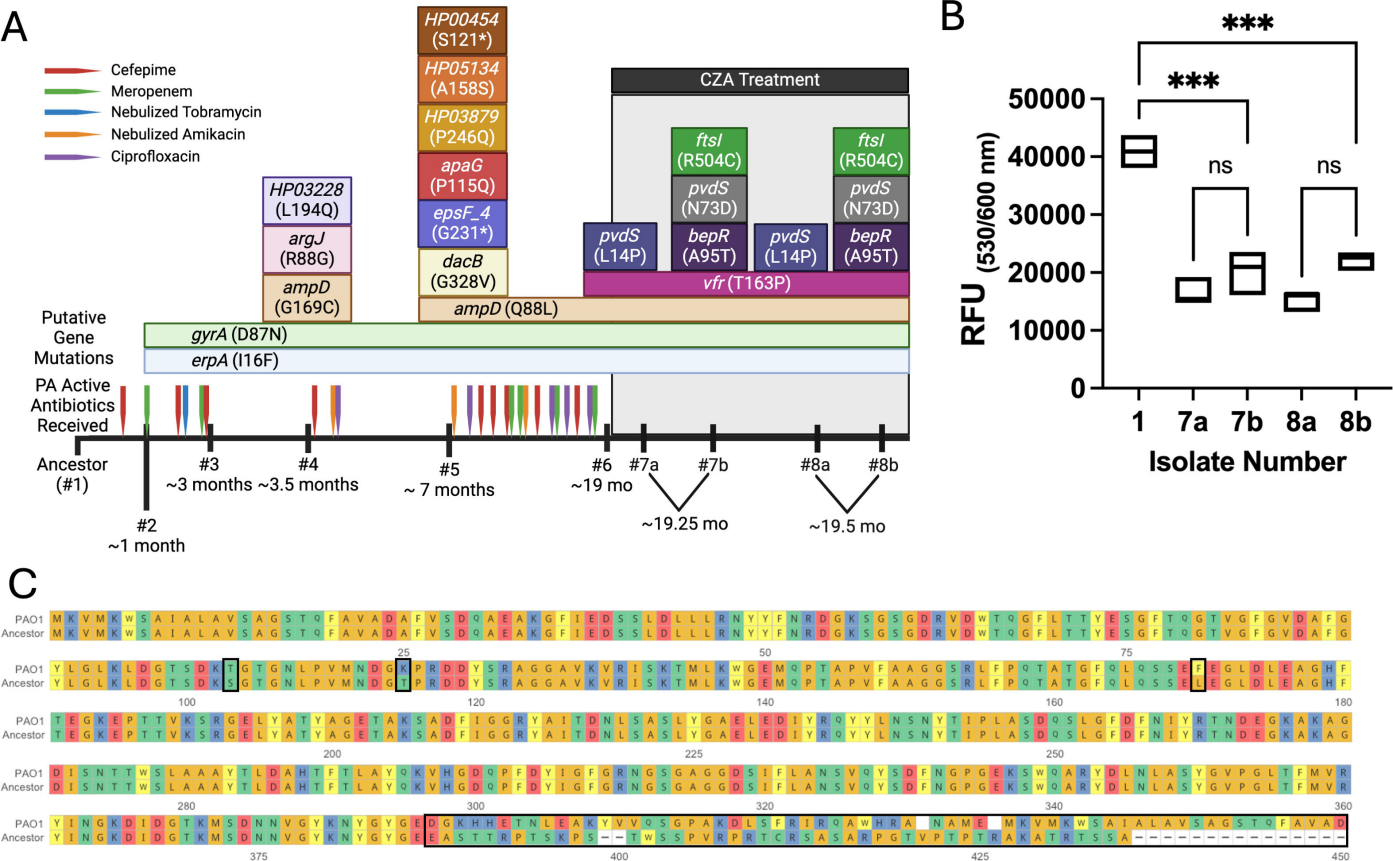
Putative mutations associated with CZA, C/T, and IMI/REL resistance do not confer increased efflux. (**A**) Timeline of accumulated mutations in clinical isolates over time. Number indicates sample date, and letter differentiates isolates from the same date. Mutations are in colored boxes overlying the tick mark on the timeline for the isolate number. Gene names are indicated in italics with amino acid substitutions in parentheses. HP in mutation boxes indicates a hypothetical protein not annotated by Prokka. CZA treatment is shown as a black bar above the mutations. Times below the isolate numbers are relative to when the ancestor isolate (#1) was collected. Flags on the timeline indicate treatment with *P. aeruginosa* active antibiotics prior to CZA treatment, and the color of the flags indicates which antibiotic was used. (**B**) Ethidium bromide efflux assay was performed using all isolates normalized to bacterial density. Fluorescence readings were taken at 1 hour after the addition of ethidium bromide and relative fluorescence units (RFUs) are reported. Significance was tested using one-way ANOVA with Tukey’s multiple comparisons test. ***P < 0.001. (**C**) Amino acid sequence alignment of OprD for ancestor as compared to PAO1 gene showing multiple mutations and frameshift mutation in OprD in the ancestor. Mutations are highlighted by black squares.

To identify mutations that could explain the CZA, C/T, and IMI/REL resistance phenotypes, we compared longitudinal isolates to the ancestor (14). We found three proteins with amino acid substitutions in CZA-, C/T-, and IMI/REL-resistant strains not present in susceptible strains: *ftsI* (R504C)*,* encoding penicillin-binding protein 3 (PBP3), *nalD* (A95T)*,* encoding a MexAB-OprM transcriptional repressor (19), and *pvdS* (N73D), encoding a virulence regulator (20) (Fig. 2A). The *nalD* gene was initially annotated by Prokka as *bepR* in the ancestral genome but was determined to be identical to *nalD* in the well-established PAO1 reference genome, thus we refer to it as *nalD*. As a transpeptidase for cell wall synthesis, the *ftsI* mutation relates directly to the mechanism of action of CZA, C/T, and IMI/REL. The mutation in *nalD* in isolates 7b and 8b is outside the likely DNA-binding domain, residues 32–51, but could influence MexAB-OprM repressor function. We tested the ancestor and evolved isolates for efflux pump activity using an ethidium bromide assay (21). Efflux was increased in all isolates compared to ancestor, indicated by lower fluorescence. The resistant isolates, 7b and 8b, had equivalent efflux to 7a and 8a (Fig. 2B), eliminating this as the primary resistance mechanism.

To determine pre-existing mutations in the ancestral genome that may influence our findings, we queried the genome of the ancestor for acquired resistance genes using ABRicate with the CARD database and 80% thresholds for both coverage and identity (22). All resistance genes noted by ABRicate search were known resistance genes in the PAO1 genome. Additionally, we compared the BL import and efflux-related genes, *oprD, mexR, nalD, cpxR, mexA, mexB*, and *oprM,* in the ancestral isolate to PAO1 reference sequences with multisequence alignments using Clustal Omega (23). Of these genes, *oprD* was the only one found to have an existing mutation in the ancestor. The *oprD* sequence differed at several positions, most notably a single nucleotide deletion leading to a frameshift mutation starting at amino acid 387 (Fig. 2C). This preexisting mutation was present in all susceptible and resistant isolates and does not explain the evolved resistance.

The development of coincident CZA, C/T, and IMI/REL resistance in this *P. aeruginosa* is best explained by the PBP3 mutation. Due to their mechanism of action, BL/BLIs provide selective pressure for mutations in penicillin-binding proteins and beta-lactamases. The PBP3 R504C substitution has previously been shown to occur in beta-lactam-resistant isolates (11, 24) and respiratory isolates from persons with cystic fibrosis (25). This mutation was previously reported in IMI/REL-resistant strains, but other mutations were also present limiting causative conclusions (10, 11, 24). With minimal confounding mutations, we conclude that this PBP mutation is necessary for cross-resistance to novel BL/BLIs. While uncommon in *P. aeruginosa* compared to efflux and porin mutations (9), PBP mutations represent a mechanism to eliminate the efficacy of multiple antimicrobials active against MDR-*P. aeruginosa*. In the resistant isolates, a mutation was found in the gene encoding NalD, a repressor of the MexAB-OprM efflux system (19). We, therefore, explored the possibility that increased efflux produced the resistant phenotype. We found that efflux levels were indistinguishable between the susceptible, 7a and 8a, and resistant, 7b and 8b, mutants, leading us to conclude that altered efflux did not drive the change in resistance.

The putative mechanism of cross-resistance provided by a SNP in PBP3 discovered here highlights the need to understand bacterial resistance mechanisms to novel antimicrobials to guide drug development and treatment for MDR organisms. We acknowledge that while this mutation was necessary to confer CZA, C/T, and IMI/REL resistance in this strain background, it may not be sufficient as there were other mutations identified in the resistant isolates. A recent study found that PAO1 with the PBP3 R504C substitution shown here did not confer increased MIC to imipenem (26). The discordant result between this study and the prior study might be explained by strain background. Our ancestral strain had an existing OprD mutation, and an AmpD mutation occurred before the PBP3 mutation and resistant phenotype (Fig. 2A). OprD is a porin that permits diffusion of carbapenems into the bacterial cells (27), thus mutations in this gene are linked to carbapenem resistance. AmpD regulates AmpC beta-lactamase expression, and AmpD mutations have been associated with AmpC overexpression (28). It is possible that AmpC overexpression combined with the PBP3 mutation resulted in CZA, C/T, IMI/REL resistance; however, this requires direct testing. The combination of the OprD and AmpD mutations, present in isolates 5, 6, 7a, and 8a, did not confer resistance to IMI/REL; therefore, we conclude that the PBP3 mutation was necessary for resistance in this background.

One limitation of this work is that the completeness of variant calling with short-read assemblies can be limited by gaps in the assembled genome. However, the high quality of the genome assembly permitted the identification of mutations that plausibly explain the cross-resistance.

Our findings provide strong evidence that novel anti-pseudomonal antibiotics can drive cross-resistance through PBP mutations. They highlight that changing antimicrobial class when resistance emerges to a novel BL/BLI during treatment may be necessary to avoid cross-resistance. Additionally, they emphasize the value of longitudinal isolate whole-genome sequencing from infections to readily identify bacterial genetic causes of treatment failure. Finally, they underscore that drug development should explore alternative antibiotic targets to prepare to circumvent future resistance to current antibiotics.

## Data Availability

All genomes from the sequenced isolates for this work will be uploaded to the NCBI Genome Database. Isolate accession numbers are SAMN43824006, SAMN43824007, SAMN43824008, SAMN43824009, SAMN43824010, SAMN43824011, SAMN43824012, SAMN43824013, SAMN43824014, and SAMN43824015.

## SUPPLEMENTAL MATERIAL

Supplemental methods spectrum.02312-24-s0001.docx Provides detailed methods for the study.

ASM does not own the copyrights to Supplemental Material that may be linked to, or accessed through, an article. The authors have granted ASM a non-exclusive, world-wide license to publish the Supplemental Material files. Please contact the corresponding author directly for reuse.
