# Supplementary material for: Mutations leading to ceftolozane/tazobactam and imipenem/cilastatin/relebactam resistance during in vivo exposure to ceftazidime/avibactam in Pseudomonas aeruginosa
Source: Microbiol Spectr. 2025 Feb 11;13(3):e02312-24. doi: 10.1128/spectrum.02312-24 (PMC11878083; doi:10.1128/spectrum.02312-24)
Supplement: Supplemental methods — Provides detailed methods for the study. [file spectrum.02312-24-s0001.docx]

SUPPLEMENTARY INFORMATION:

DETAILED METHODS

**Bacterial Isolates**

Bacterial isolates were obtained from the UPMC Children’s Hospital of Pittsburgh microbiology lab and frozen in 25% glycerol in LB at -80 ºC. Isolates were obtained from LB agar plates and an overnight culture from a single colony was grown at 37 ºC in LB broth on a roller drum at 80 rpm. Bacteria were pelleted from 1 mL of overnight culture for genomic DNA extraction.

**DNA Extraction and Sequencing**

Genomic DNA was extracted using Qiagen DNAeasy Blood and Tissue Kit following the manufacturer’s instructions. DNA concentration was measured using a Qubit fluorimeter (Life Technologies). Next generation sequencing libraries were prepared and sequenced on the Illumina platform by SeqCoast Genomics (Portsmouth NH) at 200 Mbp/1.3 million reads depth.

Per SeqCoast, sequencing was performed on the Illumina NextSeq2000 platform using a 300 cycle flow cell kit to produce 2x150bp paired reads. 1-2% PhiX control was spiked into the run to support optimal base calling. Read demultiplexing, read trimming, and run analytics were performed using DRAGEN v3.10.12, an on-board analysis software on the NextSeq2000.

**Whole Genome Sequencing Analysis and Variant Calling**

Reads were trimmed with Trimmomatic v0.36 and genomes were assembled using SPAdes v3.12.0 (1, 2). Assembly quality was assessed using Quast v5.0.2 (3). Genome annotation was performed using Prokka v1.14.16 and variant calling was completed using breseq v0.38.1 (4-7). The breseq output was manually reviewed to eliminate variant calls that likely originated from the junction of reads or contigs. This analysis did not utilize any original code.

**Phylogenetic Analysis**

Core genome assemblies were created with Panaroo v1.5.0 (8) Phylogeny was created using RAxML v8.2.11 with the rapid bootstrap algorithm, 2421 random seed, and 1000 runs (9-11) and tree was visualized using iTOL (12) (https://itol.embl.de/itol.cgi). This analysis did not use any original code.

**Minimum Inhibitory Concentration (MIC) Testing**

Isolates were struck onto LB agar plates and colonies were isolated and diluted in PBS to a McFarland standard of 0.5. Bacterial lawns were inoculated onto Mueller-Hinton agar (BD, Difco) and ceftazidime/avibactam, ceftolozane/tazobactam, and imipenem/relebactam MIC test strips (Liofilchem, Teramo, Italy) were placed onto plates prior to incubation. Plates were incubated at 37 ºC for 24 hours prior to reading MICs. MIC was deteremined at the site where the lawn contacted the MIC strip. If lawn touched the strip between lines indicating concentrations, then the higher concentration was recorded as the MIC.

**Ethidium Bromide Efflux Assay**

Efflux activity was measured with the ethidium bromide uptake assay based on previously published methods (13). Cultures were grown overnight for 18 hours, and 500 µL was pelleted and washed twice and resuspended with PBS. A black flat-bottom 96-well plate (Corning ref. 3925) was seeded with 90 µL of resuspended cells and 10 µL of 10 µg/mL ethidium bromide (Invitrogen by Thermo Fisher Scientific ref. 15585-011). Fluorescence intensity was measured 1 hour after ethidium bromide was added at an excitation of 530 nm and emission of 600 nm. High efflux activity would result in a low fluorescence intensity as less ethidium bromide is left within the cells binding to DNA. Efflux activity was measured in biological triplicate with at least three technical replicates each.

REFERENCES

1. Bankevich A, Nurk S, Antipov D, Gurevich AA, Dvorkin M, Kulikov AS, Lesin VM, Nikolenko SI, Pham S, Prjibelski AD, Pyshkin AV, Sirotkin AV, Vyahhi N, Tesler G, Alekseyev MA, Pevzner PA. 2012. SPAdes: a new genome assembly algorithm and its applications to single-cell sequencing. J Comput Biol 19:455-77.

2. Bolger AM, Lohse M, Usadel B. 2014. Trimmomatic: a flexible trimmer for Illumina sequence data. Bioinformatics 30:2114-20.

3. Gurevich A, Saveliev V, Vyahhi N, Tesler G. 2013. QUAST: quality assessment tool for genome assemblies. Bioinformatics 29:1072-5.

4. Deatherage DE, Barrick JE. 2014. Identification of mutations in laboratory-evolved microbes from next-generation sequencing data using breseq. Methods Mol Biol 1151:165-88.

5. Seemann T. 2014. Prokka: rapid prokaryotic genome annotation. Bioinformatics 30:2068-9.

6. Santos-Lopez A, Marshall CW, Haas AL, Turner C, Rasero J, Cooper VS. 2021. The roles of history, chance, and natural selection in the evolution of antibiotic resistance. Elife 10.

7. Santos-Lopez A, Fritz MJ, Lombardo JB, Burr AHP, Heinrich VA, Marshall CW, Cooper VS. 2022. Evolved resistance to a novel cationic peptide antibiotic requires high mutation supply. Evol Med Public Health 10:266-276.

8. Tonkin-Hill G, MacAlasdair N, Ruis C, Weimann A, Horesh G, Lees JA, Gladstone RA, Lo S, Beaudoin C, Floto RA, Frost SDW, Corander J, Bentley SD, Parkhill J. 2020. Producing polished prokaryotic pangenomes with the Panaroo pipeline. Genome Biol 21:180.

9. Stamatakis A. 2014. RAxML version 8: a tool for phylogenetic analysis and post-analysis of large phylogenies. Bioinformatics 30:1312-3.

10. Stamatakis A. 2006. RAxML-VI-HPC: maximum likelihood-based phylogenetic analyses with thousands of taxa and mixed models. Bioinformatics 22:2688-90.

11. Antoine J, Inglis GDT, Way M, O'Rourke P, Davies MW. 2020. Bacterial colonisation of the endotracheal tube in ventilated very preterm neonates: A retrospective cohort study. J Paediatr Child Health 56:1607-1612.

12. Letunic I, Bork P. 2024. Interactive Tree of Life (iTOL) v6: recent updates to the phylogenetic tree display and annotation tool. Nucleic Acids Res 52:W78-W82.

13. Lonergan ZR, Nairn BL, Wang J, Hsu YP, Hesse LE, Beavers WN, Chazin WJ, Trinidad JC, VanNieuwenhze MS, Giedroc DP, Skaar EP. 2019. An Acinetobacter baumannii, Zinc-Regulated Peptidase Maintains Cell Wall Integrity during Immune-Mediated Nutrient Sequestration. Cell Rep 26:2009-2018 e6.
